# The Autophagy Receptor *SQSTM1*/p62 Is a Restriction Factor of HCMV Infection

**DOI:** 10.3390/v16091440

**Published:** 2024-09-10

**Authors:** Nadine Krämer, Uxía Gestal Mato, Steffi Krauter, Nicole Büscher, Ahmad Afifi, Lina Herhaus, Luise Florin, Bodo Plachter, Christine Zimmermann

**Affiliations:** 1 Institute for Virology and Forschungszentrum Immuntherapie, University Medical Center of the Johannes Gutenberg-University, 55131 Mainz, Germany; nadine.kraemer@uni-mainz.de (N.K.); steffi.krauter@gmx.de (S.K.); bueschni@uni-mainz.de (N.B.); afifiaa@outlook.com (A.A.); lflorin@uni-mainz.de (L.F.); christine_zimmermann@gmx.de (C.Z.); 2 Institute of Biochemistry II (IBC2), Goethe University School of Medicine, Theodor-Stern-Kai 7, 60590 Frankfurt, Germany; gestalmato@em.uni-frankfurt.de (U.G.M.); lina.herhaus@gmail.com (L.H.)

**Keywords:** human cytomegalovirus, autophagy receptor, *SQSTM1*/p62, optineurin, host cell defense

## Abstract

(1) Background: Intrinsic defense mechanisms are pivotal host strategies to restrict viruses already at early stages of their infection. Here, we addressed the question of how the autophagy receptor sequestome 1 (*SQSTM1*/p62, hereafter referred to as p62) interferes with human cytomegalovirus (HCMV) infection. (2) Methods: CRISPR/Cas9-mediated genome editing, mass spectrometry and the expression of p62 phosphovariants from recombinant HCMVs were used to address the role of p62 during infection. (3) Results: The knockout of p62 resulted in an increased release of HCMV progeny. Mass spectrometry revealed an interaction of p62 with cellular proteins required for nucleocytoplasmic transport. Phosphoproteomics further revealed that p62 is hyperphosphorylated at position S272 in HCMV-infected cells. Phosphorylated p62 showed enhanced nuclear retention, which is concordant with enhanced interaction with viral proteins relevant for genome replication and nuclear capsid egress. This modification led to reduced HCMV progeny release compared to a non-phosphorylated version of p62. (4) Conclusions: p62 is a restriction factor for HCMV replication. The activity of the receptor appears to be regulated by phosphorylation at position S272, leading to enhanced nuclear localization, viral protein degradation and impaired progeny production.

## 1. Introduction

Viral infections are antagonized at different levels by host defense strategies. Adaptive immune mechanisms are highly effective in controlling infection and in preventing future encounters with the same agent by establishing immunological memory. However, these effectors are only active as a second or third line of defense, as they have to be induced in the organism, e.g., by stimulating immune cells prior to action. However, restrictive mechanisms for an immediate response to viral infection are already implemented at the level of individual cells. Many of these intrinsic mechanisms are constitutively active, but they may also be swiftly induced following infection [1,2]. An important strategy is the elimination of viral proteins via diverse pathways [3,4,5]. Autophagy has been recognized as such an intrinsic cellular defense mechanism against viruses [6,7,8]. In this process, also referred to as virophagy, autophagy receptors bind either directly to viral proteins or to biochemical tags such as ubiquitin or Small Ubiquitin-like Modifiers (SUMOs), which are conjugated to the cargo [9,10,11,12,13,14]. Subsequently, autophagy receptors recruit the cargo to autophagosomal membranes, directing the cargo to nascent autophagosomes [15]. After fusion with the lysosome, the cargo, i.e., viral proteins, are degraded. Viruses, on the other hand, have developed multiple mechanisms to evade degradation processes. Autophagy is rapidly induced following infections with the human cytomegalovirus (HCMV). Both proviral and antiviral effects of autophagy and autophagy induction have been reported, apparently representing the complex interaction of HCMV with the autophagy machinery [16,17,18,19,20,21,22].

Autophagy receptors are important players in the process of autophagosomal degra-dation of viruses and viral components. All autophagy receptors are able to selectively bind to their cargo and to promote its recruitment to the phagophore mainly by interacting with the anchored LC3 proteins [23,24]. One of the best characterized autophagy receptors is sequestome-1 (*SQSTM1*/p62), the first selective receptor characterized in mammalian cells [25,26]. *SQSTM1*/p62, hereafter referred to as p62, plays a role in various viral infections. For example, in coxsackievirus B3 (CVB3) infection, p62 targets a ubiquitinated capsid component for degradation, acting as a viral restriction factor [27,28]. Similarly, in human immunodeficiency virus (HIV) infection, p62 interacts with the viral Tat protein in an ubiquitin-independent fashion, leading to its selective degradation [29]. In HCMV infection, p62 induces the non-canonical Keap1-Nrf2 pathway, resulting in HCMV inhibition in vitro [30]. However, p62 can also support viral infection, as is the case for human papillomavirus, where it interacts with incoming capsids and accompanies the virus to ProMyelocytic Leukemia Nuclear Bodies (PML-NBs), which are the sites of viral transcription and replication [31].

In addition to its function as a cargo receptor for autophagy, p62 is involved in several important pro- and anti-inflammatory pathways, including the activation of the transcription factor Nuclear Factor ‘kappa-light-chain- enhancer’ of activated B-cells (NF-κB) in distinct contexts [32,33,34]. p62 can also transport ubiquitinated substrates to the proteasome, such as the cellular protein tau, which is involved in Alzheimer’s disease [35,36]. Another feature of p62 is its ability to shuttle between the nucleus and cytoplasm, using its nuclear localization signals (NLS) and a nuclear export signal (NES) [37,38]. Thus, p62 is a multifunctional protein and a key player in different forms of autophagy, such as virophagy, but it may restrict viral replication also on other levels.

p62 is induced following HCMV infection [17]. We have previously shown that p62 interacts with a number of HCMV proteins in late-stage infected cells [20]. It is recruited to specialized sites in the cytoplasm, termed cytoplasmic assembly compartments (cVACs), where viral tegumentation and envelopment are orchestrated. It also localizes to viral particles in the cytoplasm, as shown by immunoelectron microscopy [20]. Finally, p62 is packaged into mature, extracellular HCMV virions [20,22]. These data suggest that p62 plays an important role during HCMV infection. In this work, the role of p62 during early stages of HCMV infection was addressed. The infection of CRISPR-Cas9 knockout cells of p62 revealed enhanced viral genome replication and progeny release, indicating that p62 restricts HCMV infection already at early stages. Further analyses revealed that p62 interacted with proteins involved in nucleocytoplasmic transport and that the localization and stability of the protein was regulated by phosphorylation in HCMV-infected cells, affecting viral progeny production.

## 2. Materials and Methods

### 2.1. Plasmids

The expression plasmid pLKO5.U6.sgRNA(BsmBI, stuffer).EFS.SpCas9.P2A.tag*GFP*, enabling the gRNA cloning and CRISPR/Cas9-mediated knockdown was kindly provided by Dirk Heckl (Martin Luther University, Halle, Germany) via Melanie Brinkmann (Technische Universität Braunschweig, Braunschweig, Germany). The corresponding envelope expressing plasmid VSV-G pMD2.G (#12259, AddGene, Teddington, UK) and the packing plasmid gag-pol psAX2 (#12260, AddGene) were kindly provided by Melanie Brinkmann. For the stable expression of genes in HFFs, a modified pLKO-based lentiviral vector, as well as the lentiviral packaging plasmids pLP1, pLP2, and pLP/VSV-G, were used and kindly provided by Myriam Scherer (University of Ulm, Ulm, Germany) [2].

### 2.2. Cell Culture

Primary human foreskin fibroblasts (HFFs) and human embryonic kidney (HEK) 293 cells, expressing the simian virus 40 large t antigen (HEK293T), were cultured in T-175 cm^2^ flasks at 37 °C, 90% humidity and 5% CO_2_. HFFs were cultured in MEM with 10% FCS, 2 mM L-glutamine, 0.5 ng/mL bFGF and 50 mg/L gentamycin. HEK293T was maintained in DMEM supplemented with 10% FCS, 2 mM L-glutamine, and 50 mg/L gentamycin. For some experiments, infected HFFs were treated with 10 µM of MG132 (M7449, Merck KGaA, Darmstadt, Germany) once 18 h before cell harvest or with 2 µM of bafilomycin A1 (sc-201550A, Santa Cruz Biotechnology, Dallas, TX, USA) once 4 h before cell harvest.

### 2.3. Generation of Autophagy Receptor Knockout (ko) HFFs Using CRISPR/Cas9-Mediated Genome Editing

Customized gRNAs were designed to inhibit the respective expression of p62 and OTPN by using the website http://crispor.tefor.net/ (accessed on 9 July 2020) [39,40]. The gRNA oligonucleotides, including a forward and a reverse sequence, were purchased from Metabion International AG (Planegg, Germany) and were as follows:

g_*SQSTM1*_Fwd 5′-CACCGAATGGCCATGTCCTACGTGA; g_*SQSTM1*_Rev 5′-AAACTCACGTAGGACATGGCCATTC;

g_*OPTN*_Fwd 5′-CACCGCCTGGACACGTTTACCCCGG; g_*OPTN*_Rev 5′-AAACCCGGGGTAAACGTGTCCAGGC.

*SQSTM1*-gRNA targets exon 2 at nucleotide 67 of *SQSTM1* and the *OPTN*-gRNA directs exon 1 at nucleotide 113 of *OPTN*.

The HFF-knockout cells were generated as described by Gonzalez-Perez et al. [41]. Briefly, the gRNAs were cloned into the lentiviral vector pLKO5.U6.sgRNA(*Bsm*BI, stuffer).EFS.SpCas9.P2A.tag*GFP* (plKO5). For this, the *Bsm*BI (FD0454, Thermo Fischer Scientific, Waltham, MA, USA) linearized vector was ligated with the annealed gRNA sequences or as control with dH_2_O. The vector includes *GFP* for the selection of transduced HFFs by flow cytometry.

For the transduction of HFFs with the lentiviral pLKO5 supernatant to produce knockout cells, 5 × 10^6^ HEK293T cells were seeded in 10 cm^2^ dishes containing 10% DMEM. The next day, cells were transfected with 2.8 µg pMD2.G, 11 µg psPAX2 and 14 µg plKO5, containing either the gRNA of interest or no gRNA, with Lipofectamine 2000 Transfection Reagent (11668019, Thermo Fischer Scientific) in OptiMEM (31985070, Thermo Fischer Scientific). Eighteen hours after transfection, the medium was changed to lentiviral harvest medium (DMEM, supplemented with 20% FCS, 10 mM HEPES, 5630080, Thermo Fisher Scientific) and 50 mg/L gentamycin. Then, 36 h post-transfection, the supernatant of HEK293T was collected and filtered through a 0.45 μm filter. Afterwards, the supernatant was diluted 1:2 with HFF medium, and polybrene (sc134220, Santa Cruz Biotechnology) at a concentration of 4 µg/mL was added. The HFF medium, seeded in 6-well plates with 0.25 × 10^6^ the previous day, was exchanged with 3 mL lentivirus-containing medium. To enhance the lentiviral transduction, 6-well plates were centrifuged at 700× *g* at RT for 90 min. Cells were then incubated, and after 4 h post-transduction, the medium was replaced with HFF medium. Four days after transduction, successfully transduced cells were sorted by flow cytometry for the *GFP* signal. The *SQSTM1* and *OPTN* knockout cells, as well as control cells, were seeded. The knockout of each gene was validated by Western blot.

### 2.4. Generation of HFF GFP-SQSTM1

Permanently *GFP*-p62 expressing HFFs were generated using the lentiviral transduction method [42]. For this purpose, a nucleotide sequence encoding *SQSTM1* was designed with the *GFP*-encoding sequence inserted in frame at the 3′-end. This construct was inserted into the *Bsm*BI-linearized pLKO-based vector. For lentivirus production, 5 × 10^6^ HEK293T cells were seeded in 10× cm^2^ dishes containing 10 mL DMEM culture medium. Twenty-four hours later, the medium was removed and replaced with 5 mL DMEM culture medium absent of antibiotics. Six hours later, cells were transfected with 6 μg of pLKO-*GFP-SQSTM1* together with 3 μg each of the three packaging plasmids, pLP1, pLP2 and pLP/VSV-G complexed with Lipofectamine 2000 Transfection Reagent. Eighteen hours after transfection, the medium was replaced with DMEM culture medium. Forty-eight hours post-transfection, the harvested lentiviral supernatant was filtered through a 0.45 μM filter, 1:2 diluted with HFF medium and supplemented with 7.5 μg/mL polybrene. HFFs were seeded the day before transduction in 10 cm^2^ dishes (0.5 × 10^6^). Then, 24 h later, the HFF medium was exchanged with the medium containing lentivirus. After 24 h of incubation, the medium was replenished with fresh HFF medium. To obtain HFF-*GFP-SQSTM1*, 500 μg/mL geneticin was added to the medium two days after transduction. These cells were cultured with medium and permanently supplemented with 500 μg/mL geneticin.

### 2.5. Viruses and BAC-Mutagenesis

The following strains were used in this study: BADwt/Ad169 [43] (here designated HCMV), kindly provided by Thomas Shenk (Princeton University, Princeton, NJ, USA), TB40/E-BAC7 (here designated TB40/E), a derivate of the endotheliotropic strain TB40/E [44], kindly provided by Christian Sinzger (University of Ulm, Ulm, Germany), HCMV-p62-S272wt, HCMV-p62-S272A and HCMV-p62-S272D, which were each generated for this study. All HCMV strains and variants were derived from bacterial artificial chromosome (BAC) clones.

The cloning of each strain was based on the BAC recombineering technology described by Warming et al. [45]. To create HCMV-p62-S272wt, HCMV-p62-S272A and HCMV-p62-S272D, the non-essential gene region *UL1-6* of the parental strain BADwt was first replaced with the bacterial *galK* gene. In a second step, either the wild-type sequence of *SQSTM1* or p62 mutant sequences were re-inserted (designed sequences were ordered from Synbio Technologies, Monmouth Junction, NJ, USA). In these mutant versions, the wild-type serine (S) of S272 was replaced by either alanine (A) or aspartate (D). For gene expression, a modified version of the major immediate-early promotor (MIEP) of HCMV containing a non-functional cis repressive sequence (crs) was inserted upstream [20,46,47]. Virus reconstitution from BAC clones was obtained by transfecting BAC-DNA into HFFs with a Superfect transfection reagent (Qiagen, Hilden, Germany), as described previously [20,48].

### 2.6. Quantitative PCR Analysis

Quantitative PCR analysis was performed to analyze HCMV genome replication over time. The virus titer determination was completed as recently described [49]. For the experiment, HFFs were infected with infectious cell culture supernatants adjusted to transfer 4 genomes per cell. Cells were collected on different days post infection. Viral genomes were isolated from 1 × 10^5^ cells, using the High Pure Viral Nucleic Acid Kit (11858874001, Roche Holding AG, Basel, Switzerland) according to the manufacturer’s instructions.

The analysis was conducted in 50 µL of reaction mixture containing 5 µL of isolated DNA or serially diluted standard DNA from HCMV cosmid pCM1049 [50]. The following cycling parameters, consisting of one initial step of 10 min at 95 °C, followed by 42 amplification cycles of 15 s at 95 °C and 1 min at 60 °C, were used. Three technical replicates were evaluated using the 7500 Real-Time PCR system from PE Applied Biosystems, Weiterstadt, Germany.

### 2.7. IE1 Assay

The release of HCMV viral progeny was analyzed by staining for the expression of the immediate early 1 protein (IE1), using mAb-IE1 (p63-27) [51], kindly donated by William Britt, University of Birmingham, Birmingham, AL, USA. For this, 5 × 10^3^ HFFs were seeded into each well of a 96-well plate. The next day, the virus-containing supernatant, collected at various days post-infection, was added to the cells. At 48 h post-infection, cells were washed with PBS and fixed with 96% ethanol at room temperature. After 20 min, the cells were washed again with PBS and incubated with 50 µL of the undiluted primary antibody (p63-27) for 1 h at 37 °C.

After further washing steps, the cells were incubated with the secondary antibody, an anti-mouse IgG (horseradish peroxidase (HRP)-coupled rabbit anti-mouse IgG (H+L); P026002-2, Dako, Santa Clara, CA, USA), diluted 1:500 in 1× PBS. To visualize IE1-antibody binding, a freshly prepared 3-amino-9-ethylcarbazole (AEC)–H_2_O_2_ solution was added to each well for 1 h at 37 °C. After this incubation period, cells were once washed, covered with PBS and used for analysis. Eight technical replicates were evaluated by counting IE1-positive nuclei to determine the mean as a relative measure of infectivity.

### 2.8. Immunofluorescence

First, 5 × 10^5^ HFFs were seeded in 10 cm^2^ dishes equipped with acetone-treated cover slips for immunofluorescence staining. The next day, cells were infected with a multiplicity of infection (m.o.i.) of 0.05 or less. At 6 days post-infection (d.p.i.), the coverslips were collected and fixed overnight at 4 °C with 90% acetone. Before staining, the cover slips were washed three times with PBS containing 0.1% Triton. Afterwards, cells were probed with primary antibody, mAb-*SQSTM1*/p62 (D5E2¸8025, Cell Signaling Technology, Danvers, MA, USA) and mAB-pp150 (XPA36.14, kindly donated by William Britt). After incubation in a moist chamber at 37 °C for 1 h, the coverslips were washed with PBS/0.1% Triton. The cells were treated with secondary antibodies for detection (A11003, Thermo Fisher Scientific and ab150077, Abcam, Cambridge, UK) and incubated as before. Nuclei were stained with 4′,6-diamidin-2-phenylindol (DAPI) for 10 min. The immunofluorescence images were captured using a Zeiss Axiovert 200 M microscope fitted with a Plan-Apochromat 100×/1.4 Oil objective and Axiovision deconvolution software (version 4.7; Carl Zeiss, Jena, Germany).

### 2.9. Western Blot

One day before infection, 0.5 × 10^6^ HFFs or knockout HFFs were grown in 10 cm^2^ dishes. Cells were infected with the respective HCMV strains based on genomes per cell or m.o.i. On different days post-infection, HFFs were collected and adjusted to 1 × 10^5^ cells/10 µL SDS lysis buffer (125 mM Tris; 2% (*v*/*v*) β-Mercaptoethanol; 10% (*v*/*v*) glycerol; 2% (*w*/*v*) SDS; 1 mM EDTA, pH 8,0 and 0.005% (*v*/*v*) bromphenolblue). Either 1 × 10^5^ cells to investigate protein levels or 2 × 10^5^ cells to study the phosphorylation of proteins were separated on 10% Bolt™ Bis-Tris Plus gels (NW00100BOX, Thermo Fisher Scientific). The proteins were transferred to a 0.2 µM Immobilon-PSQ PVDF-membrane (ISEQ00010, Merck KGaA). The filter was probed with specific primary antibodies mAb-GAPDH (G87955, Merck KGaA), mAb-IE1 (p63-27), mAb-ISG15 (F-9, sc-166755, Santa Cruz Biotechnology), mAb-LC3B (D11) XP (3868S; Cell Signaling Technology), mAb-MCP (28-4), mAb-UL48a, mAB-pp150 (XPA36.14), and mAb-pp28 (41-18), which were all kindly donated by William Britt, pAb-Mx1 (PA5-22101, Thermo Fischer Scientific), pAb-Optineurin (ab23666, Abcam), pAb-POM121 (PA5-36498, Thermo Fischer Scientific), mAb*SQSTM1*/p62 [2C11] (ab56416, Abcam), and pAb-*SQSTM1*/p62 (phospho Ser272) (GTX133943-100, GeneTex, Irvine, CA, USA). Fluorescent-conjugated secondary antibodies (A10043, Thermo Fisher Scientific; 926-32212, LI-COR, Lincoln, NE, USA) were used for detection.

### 2.10. Coimmunoprecipitation Experiments (CoIP)

CoIPs with antibodies against either p62 or optineurin were performed to study the interaction partners of different autophagy receptors. Depending on the subsequent experimental setup, either 1.8 × 10^6^ or 0.5 × 10^6^ HFFs were sown. HCMV-infected cells (m.o.i. of 1) were collected at 3 d.p.i. and resuspended in lysis buffer (0.5 M NaCl, 0.05 M Tris-HCl pH = 8.5, 0.5% NP-40 alternative [492018, Merck KGaA], 1 mM DTT, 1 complete protease inhibitor cocktail tablet/10 mL [04693159001, Roche] and 1 PhosSTOP phosphatase inhibitor cocktail tablet/10 mL [4906837001, Roche]). The immunoprecipitation was completed as previously published [20,48]. Briefly, cells were lysed by ultrasound (1 × 10 s and 30% output), using a Branson Sonifier 250. Cleared cell extracts were either mixed with specific antibodies, either mAb-*SQSTM1*/p62 (D5E2) (8025, Cell Signaling Technology) or pAb-Optineurin (ab23666, Abcam), and incubated overnight at 4 °C on a rotating platform. The next day, protein-A/G magnetic beads (88803, Thermo Fischer Scientific) or IgG beads (NI01, Merck KGaA) were added for 4 h and incubated in a rotator at RT. The beads were washed three times with lysis buffer and two times with the same buffer without detergents. Immunoprecipitated samples (IPs) and lysate control were subjected to mass spectrometry (MS).

The analysis of phosphosites of p62 in dependence on HCMV infection was conducted by infecting HFF-*GFP-SQSTM1* with HCMV (m.o.i. = 1). Both infected and uninfected control cells were collected 3 d.p.i. and washed with lysis buffer. Accordingly, a *GFP*-Trap immunoprecipitation was performed. Cell lysates were incubated with *GFP*-Trap magnetic beads (gtd-20, ChromoTek GmbH, Planegg, Germany) overnight at 4 °C in a rotator. The beads were washed three times with lysis buffer, added with 8 M urea, and two times with detergent-free wash buffer and subsequently analyzed by MS.

### 2.11. MS Analysis of Interaction Partners and Phosphosites

The experiments were performed as described previously [52]. Bound proteins were incubated in SDC-buffer (2% SDC, 1 mM TCEP, 4 mM chloroacetamide, 50 mM Tris pH 8.5) for 30 min at 60 °C. Samples were diluted 1:2 with 50 mM Tris pH 8.5 prior to overnight digestion with Trypsin at 37 °C. Digested peptides were acidified with trifluoroacetic acid (TFA) (Sigma Aldrich) to inhibit trypsin, and peptides were prepared for SDB-RPS StageTip desalting. SDB-RPS StageTips were made by stacking two layers of 3 MEm-pore solid phase extraction SDB membranes into a 200 mL micropipette tip. Acidified peptides were loaded onto the SDB-RPS StageTips and washed with 0.2% (*v*/*v*) TFA. Peptides were eluted using a two-step elution with 1.25% ammonium hydroxide, 80% (*v*/*v*) ACN and then dried using a speed-vacuum concentrator (45–75 min at 60 °C). Dried peptides were stored at −20 °C. Peptides were analyzed on an Orbitrap EliteTM or Q Exactive HF mass spectrometer (ThermoFisher).

Raw data were analyzed using MaxQuant 1.6.5.0 with standard settings and activated LFQ quantification. The database used to identify the viral peptides was the HCMV reference protein database (UniProt), or the human reference protein database (UniProt) to identify human phosphorylation sites, and the false discovery rate (FDR) was set to 1% on protein, peptide spectrum match (PSM) and site decoy level. Statistical analysis was conducted with Perseus 1.6.5. Proteins were defined as interactors if they passed a 5% corrected one-sided two-sample Student’s *t*-test with a minimal enrichment factor. For interactors, the matrix of significant hits from the right-sided Student’s *t*-test was imputed and analyzed for variance between groups through a one-way ANOVA. Tukey’s HSD post hoc test was used to identify significant pairs. Similarly, phosphorylation sites were searched, and statistical analysis was conducted with Perseus 1.6.5. Sites were selected as significant if they had a log2 ≥ 1 and *p*-value ≤ 0.05.

### 2.12. Statistical Analysis

Statistical analyses were performed using GraphPad Prism 8, version 8.3.0 (GraphPad Software Inc., San Diego, CA, USA).

## 3. Results

### 3.1. CRISPR/Cas9 Knockout of p62 Leads to Moderately Enhanced Viral Genome Replication and Enhanced Progeny Release

**Figure 1:**
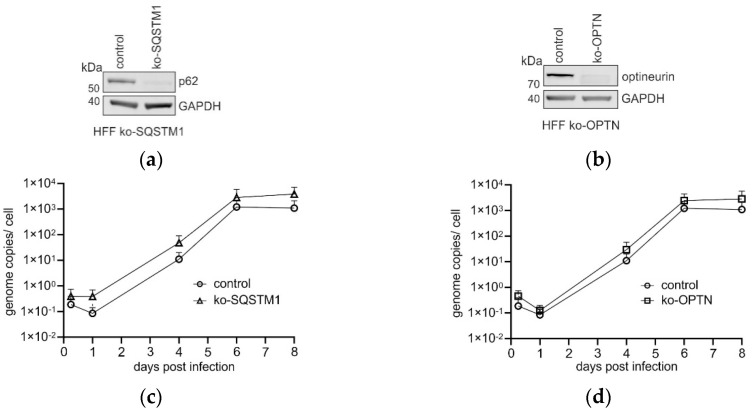

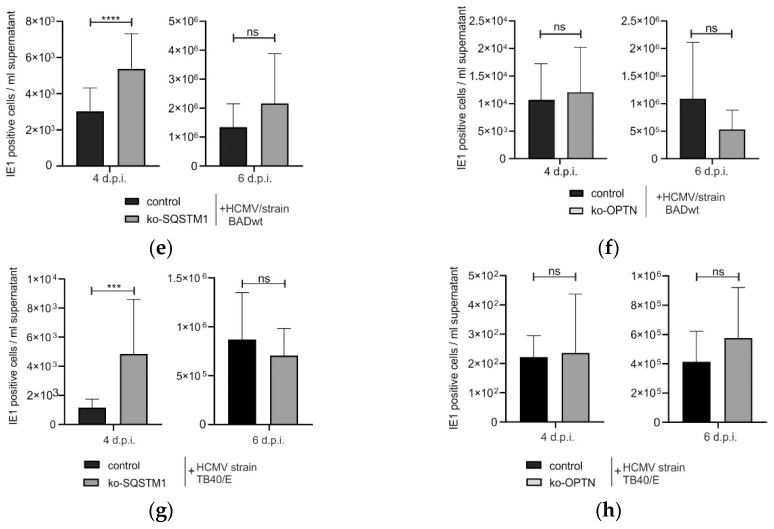
Impact of p62 and optineurin on HCMV infection. (**a**,**b**) Immunoblot analysis of HFF ko-*SQSTM1* and HFF ko-*OPTN*, using antibodies against p62, optineurin and GAPDH. (**c**,**d**) Quantitative PCR-analysis of HCMV genome replication, following infection of HFF ko-*SQSTM1* (**c**) or HFF ko-*OPTN* (**d**). Cells were infected with 4 genome copies/cell and collected at the indicated time points post-infection. Genome copies of the isolated viral DNA were determined by TaqMan qPCR. Each value represents the mean of triplicate determinations from three independent experiments. The corresponding standard deviation (SD) is represented by an error bar. (**e**,**f**) Quantitation of viral progeny release by the IE1 assay, following infection of HFF ko-*SQSTM1* (**e**) or HFF ko-*OPTN* (**f**) with HCMV (strain BADwt). The data represent mean values + standard deviations (SD) of eight technical replicates from four (HFF ko-*SQSTM1*) or three (HFF ko-*OPTN*) individual experiments for each cell line and the corresponding time point. (**g**,**h**) Quantitation of viral progeny release by the IE1 assay, following infection of HFF ko-*SQSTM1* (**g**) or HFF ko-*OPTN* (**h**) with HCMV (strain TB40). The data represent mean values + standard deviations (SD) of eight technical replicates from three individual experiments for each cell line and the corresponding time point. The statistical analysis was performed by utilizing Welch’s *t*-test. Not significant (ns): *p* > 0.05. ***: *p* ≤ 0.001. ****: *p* ≤ 0.0001.

p62 is a multifunctional adaptor protein. We could show in previous studies that p62 interacts with viral structural components in late-stage HCMV-infected cells and associates with viral capsids and viral cytoplasmic assembly compartments (cVACs) [20]. Another receptor, optineurin, was also recruited to cVACs in late-stage HCMV-infected fibroblasts [53]. Thus, knockout HFFs for p62 (HFF ko-*SQSTM1*) and for optineurin (HFF ko-*OPTN*) were established by the CRISPR-Cas9 technology (Figure 1a,b). The influence of both p62 and optineurin on viral genome replication was addressed by infecting either ko-*SQSTM1*, ko-*OPTN*, or control cells with HCMV, using an infectious dose of four genome copies/cell. Infected cells were collected at different time points after infection. Subsequently, viral DNA was isolated, and quantitative PCR was performed to determine the number of genome copies. Interestingly, a subtle but reproducible enhancement of HCMV genome replication became apparent when viral DNA levels from infected HFF ko-*SQSTM1* were compared to those from control HFFs (Figure 1c) as confirmed in four independent experiments. However, no alteration was seen in infected HFF ko-*OPTN* (Figure 1d), which was analyzed in three individual experiments. To investigate if the lack of p62 or optineurin had an impact on HCMV progeny release, HFF ko-*SQSTM1* or HFF ko-*OPTN* were infected again (m.o.i. of 0.5). The culture supernatants were collected at 4 d.p.i. and 6 d.p.i. The amount of infectious virus per volume of supernatant was analyzed using the IE1-titration assay. Significantly more progeny was released from HFF ko-*SQSTM1* at 4 d.p.i. compared to the HFF controls. A similar tendency was seen 6 d.p.i. yet not reaching statistical significance (Figure 1e). In contrast, no difference in viral progeny release was seen when supernatants from HFF ko-*OPTN* and control HFFs were compared (Figure 1f). Comparable results were obtained when a different HCMV strain (TB40E) was used for infection (Figure 1g,h). The data showed that the absence of p62 in HCMV-infected cells led to an enhancement of progeny release, whereas optineurin deficiency did not seem to have an impact.

### 3.2. p62 Interacts with a Number of Early HCMV Proteins

**Figure 2:**
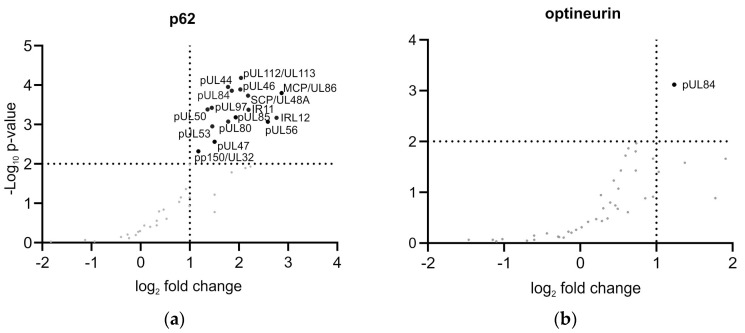
MS analysis of the interaction of p62 (**a**) and optineurin (**b**) with HCMV proteins. HFFs, infected with HCMV (m.o.i.= 1) were incubated with protein A/G magnetic beads and specific antibodies against either p62 or optineurin or IgG as control. Precipitates were analyzed and quantified by MS. They were considered significant under the following conditions: one-sided two-sample Student’s *t*-test with a minimal enrichment factor of 2 (log2(2) = 1), showing the log2 fold change and *p* < 0.01 (−log10(0.01) = 2). Hits in the upper right quarter are considered significant. All data are deposited at the PRIDE repository (PXD055196).

Previous investigations have shown that p62 interacts with viral structural proteins in late-stage HCMV-infected HFFs [20]. The analysis of the viral genome replication, however, suggested that p62 also had an early impact on HCMV infection. To address the interaction of p62 with viral proteins expressed in the early phase of infection, co-immunoprecipitation (Co-IP) experiments followed by MS analyses were performed. For this, wt-HFFs were infected with HCMV for three days and subsequently lysed. p62 was precipitated with a specific antibody. Associated proteins were analyzed by MS. For control reasons, a Co-IP experiment, using an optineurin-specific antibody, was performed in parallel. In accordance with the results of genome replication analyses, viral proteins associated with the viral DNA polymerase complex (such as pUL44, pUL84, and pUL112/113) were found to interact with p62 (Figure 2a). In addition, proteins essential for the process of nuclear egress of viral capsids, such as pUL50, pUL53, or pUL97, were co-immunoprecipitated with p62. In contrast, only pUL84 was precipitated by using an optineurin-specific antibody (Figure 2b).

### 3.3. Impact of p62 on the Steady-State Levels of Viral Proteins

**Figure 3:**
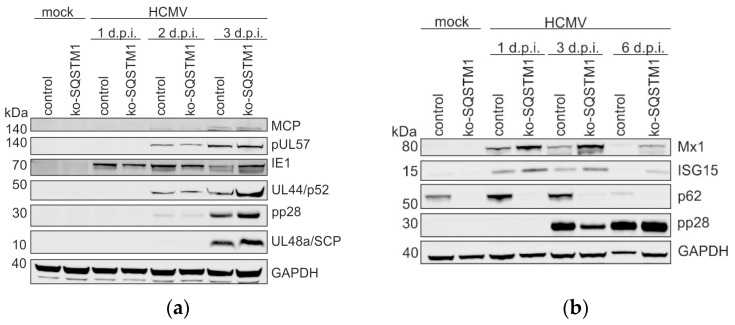
Western blot analysis of viral protein expression in HFF ko-*SQSTM1*. (**a**) Viral protein levels in dependence of p62 in HCMV-infected cells. HFF ko-*SQSTM1* and control cells were infected with HCMV strain TB40, using 8 genome copies/cell. At 1, 2 and 3 d.p.i., cells were collected and subjected to Western blot. The membrane was probed with antibodies against the viral proteins major capsid protein (MCP), pUL57, pUL44, pp28, and pUL48a. GAPDH levels were used as a loading control. (**b**) ISG levels in dependence of p62 in HCMV-infected cells. HFF ko-*SQSTM1* and control cells were infected with HCMV, using an m.o.i. of 0.5. At 1, 3 and 6 d.p.i., cells were collected and subjected to Western blot. The membrane was probed with antibodies against the ISGs proteins Mx1 and ISG15. Antibodies against p62 were used as controls. The detection of pp28 served as a control for HCMV infection. GAPDH levels were used as a loading control.

The lack of p62 had an enhancing effect on viral genome replication and progeny release, and it interacted with a number of viral early and late proteins. Thus, the question was addressed regarding whether the lack of p62 had an impact on the steady-state levels of viral proteins. For this, HFF ko-*SQSTM1* and control HFFs were again infected with HCMV. Cells were collected at 1, 2, and 3 d.p.i. Cell lysates were subjected to immunoblot analyses, using specific antibodies directed to different viral proteins, representing different phases of the replication cycle. The levels of the different viral proteins were clearly enhanced at 3 d.p.i. in cells, lacking p62 (Figure 3a), which was concordant with the antiviral effect of the latter protein. Surprisingly, however, lower levels of these proteins were found in the same HFF ko-*SQSTM1* at 1 d.p.i. and 2 d.p.i. Thus, it appeared that a mechanism restricting HCMV protein expression at these early time points of the replication cycle in HFF ko-*SQSTM1* was responsible. HCMV is known to induce type I interferon responses in fibroblasts upon infection. On the other hand, an excessive induction of type I interferon responses is abrogated by the degradation of interferon-stimulated proteins through autophagy [54,55]. We thus tested the hypothesis that the lack of p62 would enhance the levels of interferon-stimulated proteins, limiting HCMV protein expression at early times of HFF ko-*SQSTM1* infection. Thus, infected cell lysates were subjected to Western blot analysis, probing the filters with antibodies against the interferon-stimulated proteins Mx1 and ISG15 (Figure 3b). Concordant with the assumption, significantly higher levels of both proteins were detected after the HCMV infection of HFF ko-*SQSTM1* at 1 d.p.i. and 2 d.p.i. compared to infected wt cells. ISG levels were declining at later times of infection. Thus, a possible explanation for the lower viral protein levels at 1 d.p.i. and 2 d.p.i. in the absence of p62 is an enhanced interferon response leading to silenced viral protein expression. Since we could only look at two ISGs, this has to be confirmed in further detailed analyses. Irrespective of this, the lack of p62 eventually leads to an enhancement of viral productivity (see Figure 1e).

### 3.4. The Lack of p62 Has No Impact on the Induction of Autophagy in HCMV-Infected Cells

**Figure 4:**
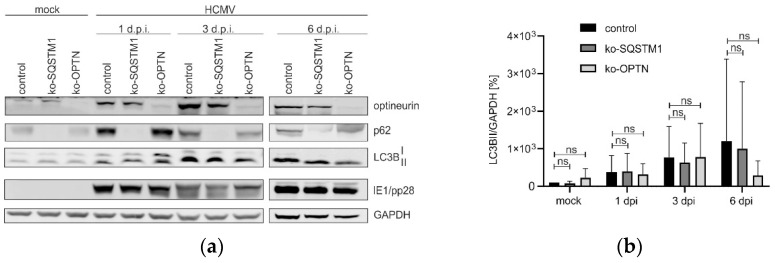
Western blot analysis of the influence of p62 and optineurin on autophagy induction. (**a**) HFF ko-*SQSTM1*, HFF ko-*OPTN*, and control cells were infected with HCMV, using an m.o.i. of 0.5. At 1, 3 and 6 d.p.i., cells were collected, and autophagy was analyzed by Western blot. LC3B was used as an indicator of autophagy functions. The turnover of the cytosolic form (LC3B-I) to the autophagosomal membrane associated from (LC3B-II) was detected with an LC3B-specific antibody. Antibodies against p62 and optineurin were applied for control. Detection of the viral IE1 protein at 1 and 3 d.p.i. and pp28 at 6 d.p.i. served as control for HCMV infection. The levels of GAPDH were used as loading control. Shown is a representative Western blot out of four experiments. (**b**) Ratio of LC3BII to GAPDH from four biological replicates. The statistical difference between each ko-*SQSTM1* and ko-*OPTN* was analyzed with two-way ANOVA with Sidak’s multiple comparisons test, ns: not significant.

HCMV infection leads to the induction of autophagy, as evidenced by increased levels of LC3B-II. To test whether the lack of p62 had an impact on the induction of autophagy by HCMV, cells were infected, and the levels of LC3B-II were measured at different time points after infection by Western blot (Figure 4a). These LC3B-II levels were quantified using four independent biological replicates and subjected to statistical analysis (Figure 4b). The lack of p62 or optineurin, respectively, had no statistically significant impact on HCMV-induced LC3B levels compared to control cells.

### 3.5. The Phosphorylation Status of p62 Is Altered Only at Serine 272 in HCMV-Infected Cells

**Figure 5:**
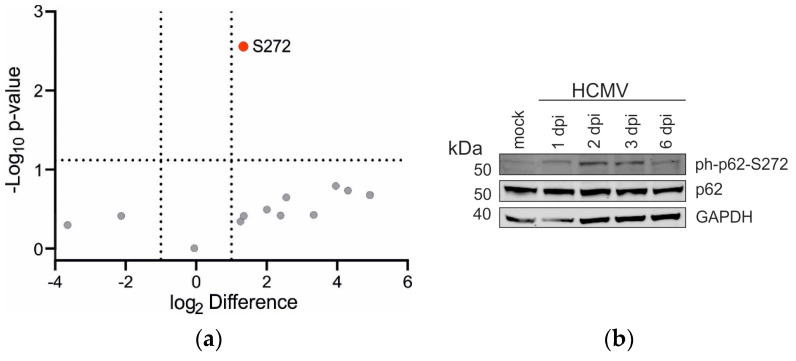
Determination of the phosphorylation status of p62 during HCMV infection. (**a**) MS analysis of the phosphorylation status of p62 in HCMV-infected versus non-infected HFF-*GFP-SQSTM1*. The detected phosphorylation sites of p62 of three biological replicates are displayed in a volcano plot, showing the fold change (X-axis) and significance as −log10 *p*-value (Y-axis). The phosphorylation sites are shown as individual data points. Changes in the phosphorylation status at S272 (colored dot) in infected versus non-infected cells reached significance above the threshold for the fold change of log2 ≥ 1 and *p*-value-log10 ≤ 0.05, which was represented by the vertical and horizontal dotted lines, respectively. Phosphorylation sites that were detected but did not show significant differences are shown as gray data points. (**b**) Validation of the phosphoproteomic data in B was performed by Western blot, using a phospho-specific antibody against p62-S272. Lysates of HCMV-infected normal HFF cells were submitted to SDS-PAGE, which was followed by Western blot analysis. The phosphorylation level of p62 at S272 was analyzed at 1, 3, and 6 d.p.i. GAPDH levels were used as loading control. One Western blot of two individual analyses is shown.

p62 interacts with selected viral proteins. This leads to reduced protein levels of the interaction partners. It is well established that the interaction of p62 with substrates is regulated by phosphorylation. The phosphorylation status of p62 in HCMV-infected cells was thus addressed by mass spectrometry (MS; Figure 5). To assess the confidence of the identification, the posterior error probability (PEP) and the modification localization probability were estimated, and the results are depicted in Table 1. Only one residue of p62, serine 272, was hyperphosphorylated upon HCMV infection (Figure 5a and Table 1), whereas the phosphorylation status of other known p62 sites remained unaffected. The p62 S272 phosphorylation was validated by an immunoblot, using a phospho-specific (S272 p62) antibody (Figure 5b). All data are deposited at the PRIDE repository (PXD055194).

The PEP value represents the probability that an observed peptide spectrum match (PSM) is incorrect; the lower the value, the higher the confidence. Similarly, the modification localization probability (also called the false localization rate) represents the probability that a given residue carries the identified modification; values above 75% (0.75) are considered highly confident.

### 3.6. Generation of Recombinant HCMV Strains, Expressing Different p62 Versions

**Figure 6:**
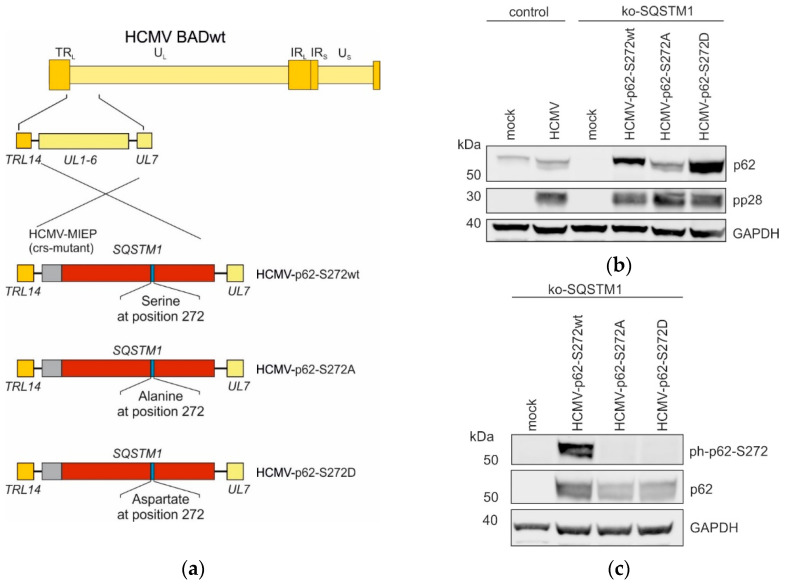
(**a**) Construction of different HCMV strains expressing p62-S272 mutants. The BAC technology was used to generate the mutant strains. All recombinant viruses were based on the HCMV parental strain BADwt. The gene region *UL1-6* of the parental strain was replaced by the *SQSTM1* gene, encoding the different mutations at the position 272 of SQSTM/p62 [serine (wt), alanine (A), or aspartate (D)]. The BACmids were reconstituted by transfection into HFFs, resulting in the viruses HCMV-p62-S272wt, HCMV-p62-S272A, and HCMV-p62-S272D. The expression of the gene was driven by the modified HCMV major immediate-early promotor (MIEP) with a non-functional cis-repressive sequence (crs) to allow a permanent expression of the respective *SQSTM1* genes in infected cells. (**b**,**c**) Western blot analysis of the p62 levels in ko-*SQSTM1* cells infected with different HCMV-*SQSTM1*-S272 strains. (**b**) Analysis of p62 levels in ko-*SQSTM1* cells, infected with the different HCMV-*SQSTM1*-S272 strains (wt/A/D). HCMV-infected wild-type (wt) HFFs were used as a control for p62. Lysates of 5 d.p.i. infected cells were collected and analyzed by Western blot with an antibody directed against p62. Viral pp28 levels were used as infection control, GAPDH levels were used as loading control. One representative Western blot from two analyses is shown. (**c**) Analysis of the levels of phosphorylation of p62 at position 272, following infection of ko-*SQSTM1* cells. Cells were infected as in (**a**) and harvested at 5 d.p.i. Samples were probed for the phosphorylation level of p62 at position 272, using a phospho-specific antibody. A representative Western blot from two analyses is shown.

To investigate whether the phosphorylation at S272 has an impact on HCMV infection, viral mutants were generated that expressed either wt-p62, a non-phosphorylated S272A version of p62, or a S272D-mutated version of p62, mimicking phosphorylation (Figure 6a). The recombinant viruses were used to infect HFF ko-*SQSTM1*. The expression of the different versions of p62 was verified by the immunoblotting of cell lysates from these cells (Figure 6b). Notably, expression levels were different, showing that the steady-state levels of p62-S272A were considerably lower than those of HCMV-p62-S272wt and HCMV-p62-S272D. The absence of phosphorylation of p62 at S272 was also verified by using a phospho-specific antibody (Figure 6c). The levels of HCMV-p62-S272D were lower compared to HCMV-p62-S272wt, which may be due to variations in infection conditions.

### 3.7. p62 Specifically Interacts with Nuclear Pore Proteins in HCMV-Infected Cells

**Figure 7:**
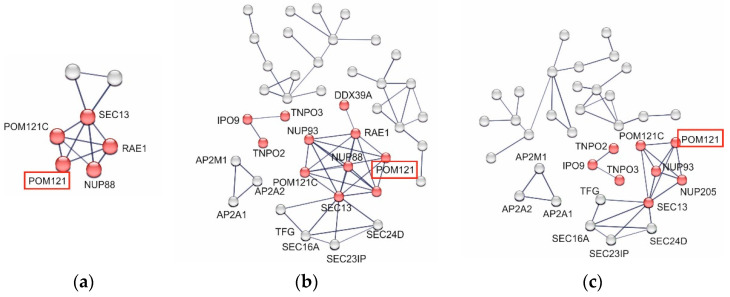
(**a**–**c**) Protein–protein interaction networks of the cellular interactors of p62 in dependence on the phosphorylation status at S272, analyzed in samples from co-immunoprecipitation analysis of HCMV-infected HFFs, using the STRING database (http://string-DBs.org, accessed on 10 August 2023). HFFs were infected with either HCMV-p62-S272wt, HCMV-p62-S272A, or HCMV-p62-S272D, respectively, using an m.o.i. of 0.5. Cells were collected at three d.p.i. p62 was immunoprecipitated using a receptor-specific antibody. The amount of p62 used for IP was adjusted to control for reduced steady-state levels of HCMV-p62-S272A-infected cells by using twice the number of lysed cells for IP against p62-S272A compared to p62-S272wt or p62-S272D. Each node represents a protein, and the red nodes represent a cluster of proteins, which are associated with the nuclear pore complex or with nucleocytoplasmic transport. The interaction network was generated with a high confidence interaction score (0.7). Networks of proteins that co-precipitated with p62-S272wt are shown in (**a**), those co-precipitated with p62-S272A are shown in (**b**), and those co-precipitated with p62-S272D are shown in (**c**).

It is well established that the affinity of p62 to its cargo proteins is regulated by phosphorylation [10]. In order to analyze whether S272 phosphorylation had an impact on the binding of p62 to cellular proteins, MS was performed. Samples were analyzed by liquid chromatography (LC)-MS, and the raw data were normalized to the respective WT and mutant p62 levels. All data are deposited at the PRIDE repository (PXD055195). Gene ontology (GO) enrichment was performed by using the Search Tool for the Retrieval of Interacting Genes (STRING) database (http://string-DBs.org, accessed on 1 August 2023) [56]. A cluster related to the functional category of nucleocytoplasmic transport was identified for each receptor variant (Figure 7). This finding is consistent with the known role of p62 in nucleocytoplasmic transport processes [57,58]. Interestingly, the nuclear envelope pore membrane protein POM 121 (POM121) was found in the precipitates from all three samples, indicating a specific interaction with p62 independent of S272 phosphorylation. POM121 is a transmembrane protein of the nuclear pore complex (NPC). The protein plays an important role in initiating NPC assembly by anchoring the complex to the nuclear envelope membrane [59,60].

### 3.8. S272 Phosphorylation of p62 Shifts the Localization of the Receptor toward the Cytoplasm

**Figure 8:**
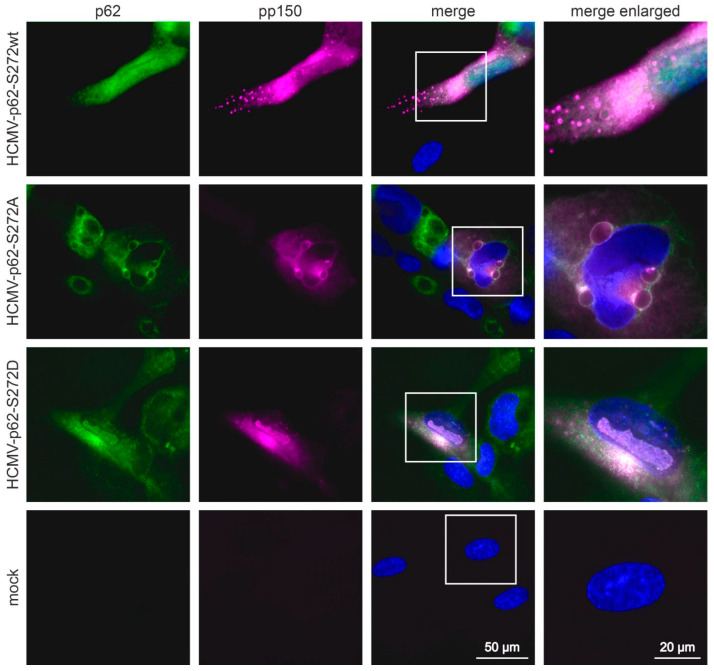
Indirect immunofluorescence analysis of the localization of p62 in infected cells, depending on S272 phosphorylation. Cells were infected for six days and stained with antibodies against p62 (green) and pp150 (purple). DAPI was used to stain nuclei (blue).

p62 is shuttling between the nucleus and the cytoplasm [57,58]. The phosphorylation of the receptor close to its nuclear localization signal at threonine 269 and serine 272 leads to increased nuclear retention, as shown in transient transfection experiments [10,57]. To address whether the phosphorylation of p62 at S272 also had an impact on the localization of the protein in HCMV-infected cells, indirect immunofluorescence analyses were carried out. HFF-ko-*SQSTM1* cells were infected with either HCMV-p62-S272wt, HCMV-p62-S272A, or HCMV-p62-S272D, respectively. At 6 d.p.i., cells were fixed and stained with antibodies against p62 and against the large viral tegument protein pp150 (Figure 8). p62 was found both in the cytoplasm and the nucleus, following infection with HCMV-p62-S272wt or HCMV-p62-S272D. In contrast, an almost complete cytoplasmic retention was seen after infection with HCMV-P62-S272A. This shows that S272 phosphorylation is sufficient to shift p62 toward the nucleus in HCMV-infected cells.

### 3.9. Lack of S272 Phosphorylation of p62 Leads to Its Proteasomal Degradation in Infected Cells

**Figure 9:**
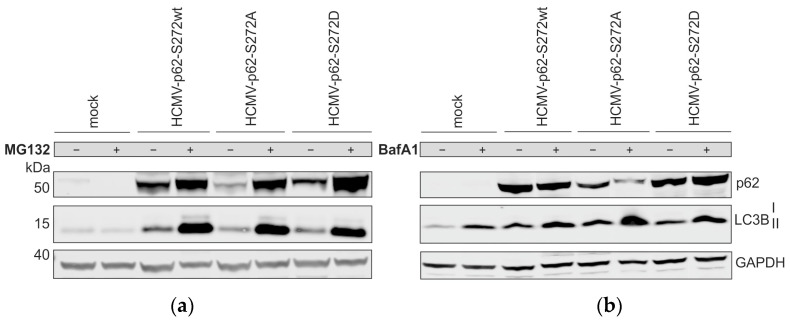
Western blot analysis of the impact of S272 phosphorylation on the proteasomal and autolysosomal degradation of p62. (**a**) Western blot analysis of p62 levels, following 5-day infection of ko-SQSTM1 cells with respective HCMV-p62-S272 strains (wt/D/A) using an m.o.i. of 0.2. Cells were treated with 10 µM of the proteasomal inhibitor MG132 18 h before sample collection. The results of one of two individual experiments are shown. (**b**) Western blot analysis of p62 levels applying 200 µM of the lysosomal inhibitor bafilomycin A1 (BafA1) 4 h before cell harvest, *n* = 1. (**a**,**b**) Cell lysates were probed with antibodies against p62, GAPDH (loading control), and LC3B.

Infection with HCMV-p62-S272A resulted in lower steady-state levels of the receptor in comparison to the two other versions. To address whether the lower steady-state levels were due to enhanced proteasomal degradation, infected HFF ko-*SQSTM1* cells were treated with the proteasome inhibitor MG132 and subjected to Western blot analysis. The levels of p62-S272A detected in MG132-treated, infected cells were comparable to the levels seen in p62-S272wt-infected and p62-S272D-infected cells without MG132 (Figure 9a). This showed that the reduced levels of p62-S272A in infected cells were a result of enhanced proteasomal degradation of the mutated protein rather than due to altered protein expression. The inhibition of autophagy by using the inhibitor bafilomycin A1 did not result in the rescue of p62 levels, indicating that the S272 phosphorylation of p62 did not impact its degradation by the autophagosome in HCMV-infected cells (Figure 9b).

### 3.10. Lack of p62-S272 Phosphorylation Leads to Enhanced HCMV Protein Expression, Genome Replication, and Progeny Release

**Figure 10:**
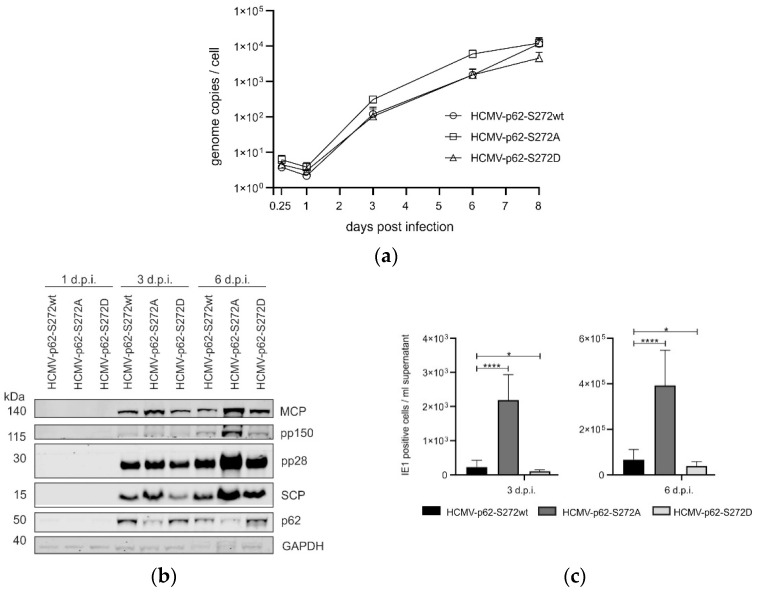
Analysis of HCMV protein expression, genome replication and progeny production in dependence on p62-272 phosphorylation. HFF-ko-SQSTM1 cells were infected with HCMV-p62-S272wt, HCMV-p62-S272A, and HCMV-p62-S272D strains, respectively, using an m.o.i. of 0.1. Cells and cell culture supernatants were collected at the indicated time points. (**a**) Quantitative PCR analysis of HCMV genome replication. The mean values of three technical replicates from three independent experiments are shown for each virus and time point. The corresponding SD is displayed as error bars. (**b**) Western blot analysis of the intracellular levels of selected viral proteins, following infection. Cells were harvested at 1, 3, and 6 d.p.i., lysed, and probed by Western blot, using antibodies against HCMV proteins MCP, pp150, pp28 and SCP. The levels of GAPDH were used as loading control. One out of two individual experiments is shown. (**c**) Viral progeny release, which is measured by the IE1 assay. The graph represents mean values of eight technical replicates from three independent experiments for each virus and time point. The mean values and corresponding SD are represented in a bar chart with error bars. Statistical analysis was performed utilizing Brown–Forsythe and Welch one-way ANOVA (not significant (ns): *p* > 0.05; *: *p* < 0.05. ****: *p* < 0.0001).

To address the impact of S272 phosphorylation on HCMV infection, viral genome replication, viral protein expression and progeny release were analyzed. HFF ko-*SQSTM1* cells were infected with p62-S272 mutants, using an m.o.i. of 0.1. Cells were harvested at six hours up to eight days post-infection. The viral DNA that was isolated from the cells was quantified by TaqMan qPCR. There was a delay in viral genome replication for HCMV-p62-S272D and HCMV-p62-S272wt compared to infection with HCMV-p62-S272A, indicating enhanced viral genome replication (Figure 10a). To address the impact on viral proteins, Western blot analyses were performed (Figure 10b). Enhanced levels of the tested viral proteins were found in HCMV-p62-S272A-infected cells in comparison to the other viruses. This showed that also the levels of viral proteins were affected by S272 phosphorylation, which is concordant with the enhanced genome replication. To address progeny release, cells were infected using the same m.o.i. Culture supernatants were collected at 3 and 6 d.p.i. and subjected to analysis by the IE1-titration assay. Progeny release from cells infected with HCMV-p62-S272A was strongly increased relative to HCMV-p62-S272wt- or HCMV-p62-S272D-infected cells (Figure 10c). Taken together, these results showed that S272 phosphorylation had a distinctly inhibitory influence on HCMV lytic infection.

## 4. Discussion

p62 is a multifunctional adaptor protein involved in many cellular processes. By interacting with small modifiers like ubiquitin, it binds to a variety of different substrates and regulates various pathways involved in inflammation, cell homeostasis and cancer development (reviewed in [32]). p62 is also involved in viral infections, playing either pro- or antiviral roles [31,61,62]. However, its function during HCMV infection is only poorly understood. p62 interacts with a number of viral structural proteins at late stages of HCMV infection [20]. p62 is targeted to viral assembly compartments in the cytoplasm and is also found in purified HCMV particles. This argues in favor of a role of p62 in particle morphogenesis [20,22]. However, it appears that p62 is also involved in other processes in the course of HCMV infection.

A slight but reproducible elevation of viral genome replication was found in HFF ko-*SQSTM1* cells compared to control HFFs. p62 thus appears to restrict HCMV infection at an early stage. In line with this, viral progeny release was enhanced. This was confirmed by the experiments, using p62-expressing viruses, where reduced levels of p62 had a marked, enhancing effect on viral progeny production (Figure 10c). One likely explanation for the phenotype seen in HFF ko-*SQSTM1* cells was an impairment of autophagy in the absence of the receptor. p62 controls autophagy via its expression level in various cell lines [63]. However, no differences were observed when LC3B-II levels were compared between infected HFF ko-*SQSTM1* cells and wt-HFFs. This indicates that p62 does not regulate autophagy in HCMV-infected fibroblasts and, more interestingly, the impact of p62 on HCMV infection in these cells is independent of its role in autophagy regulation.

MS analyses revealed that p62 interacted with HCMV early nuclear proteins, which are involved in genome replication and in nuclear capsid egress. In line with this, the steady-state levels of viral immediate-early and early proteins were reduced at 3 d.p.i. in infected wt-HFFs compared to infected HFF ko-*SQSTM1* cells. This indicates that p62 was involved in the degradation of viral proteins. p62 has been shown to deliver polyubiquitinated cargo to proteasomal degradation [36,63]. Interestingly, many of the proteins that interacted with p62 were viral nuclear polypeptides. p62 also preferentially bound to components of the nuclear pore complex. p62 is known to shuttle between the cytoplasm and the nucleus [35,57]. Thus, it is likely that p62 transports nuclear viral proteins to the cytoplasm for degradation. It remains to be determined which of the metabolic pathways for protein degradation are involved.

The induction of ISGs Mx1 and ISG15 was markedly enhanced following HCMV infection of HFF ko-*SQSTM1* cells compared to controls. p62 is involved in the regulation of IFN responses in order to prevent sustained IFN-I pathway signaling and excessive ISG synthesis [36,54,55,63]. A dependence between p62 expression and ISG induction was also reported following infection with the murine CMV and with Herpes simplex virus [55]. These authors suggested that STING degradation was mediated by p62 as a control mechanism. The results obtained in this study indicate that the interferon response is controlled by p62 also in HCMV-infected cells, which is likely to provide a balanced reaction of the cell towards the infection. The mechanisms by which this is orchestrated remain to be elucidated.

A recent publication showed that the siRNA-mediated knockdown of p62 reduced HCMV genome release [21]. We found no effect on viral genome release into the supernatant of HFF ko-*SQSTM1* compared to control HFFs. However, we did find a reproducible enhancement of viral progeny release in HFF ko-*SQSTM1*. The reason for this discrepancy with the result of König and colleagues is unclear at this point. Using the release of viral genomes is only a surrogate for viral progeny production, as it does not account for the particle-to-infectivity ratio. The divergent results may, in addition, be based on the multifunctional role of p62, which regulates cellular processes with divergent outcomes. p62 has multiple functions such as controlling apoptosis or involvement in several signal transduction cascades upon HCMV infection [30,64,65,66,67]. Thus, the permanent suppression of p62 expression in knockout cells may have a different impact on the various effects of the protein compared to cells in which the protein is knocked down. Thus, the different effects seen in this work and in the work from König et al. are likely subject to variation, depending on the methodologies and the cell lines used for analyses.

The biological activities of p62 are regulated by phosphorylation [10,32,68,69]. This work demonstrated that the only site of p62 that is hyperphosphorylated following HCMV infection is S272. Viral progeny release from HCMV-p62-S272D- and HCMV-p62-S272wt-infected cells was up to 5–10-fold lower compared to HCMV-p62-S272A infected cells. This indicated that phosphorylation at S272 of p62 had a marked restricting effect on the replication cycle of HCMV. Immunofluorescence analyses revealed a cytoplasmic retention of the unphosphorylated p62-S272A version of the receptor. The metabolic stability of this protein was, in addition, reduced by enhanced proteasomal degradation. This indicates that the lack of phosphorylation at S272 impairs nuclear import of the protein and likely its shuttling function, leading to its enhanced degradation and enhanced viral replication. In contrast, phosphorylation at S272 leads to reduced levels of nuclear viral proteins and reduced viral progeny production. Interestingly, p62 interacted with a number of proteins involved in nucleocytoplasmic transport. Phosphorylation at S272 may thus be a mechanism implemented by the cell to enhance the interaction of p62 with nuclear viral proteins in a process of shuttling these proteins to the cytoplasm for degradation.

In summary, p62 interacts with HCMV at multiple levels in infected cells, thereby interfering with viral productivity. In addition to a putative role in autophagy at late stages of HCMV infection, it also targets early processes by destabilizing viral proteins. In the latter process, the phosphorylation of p62 at S272 appears to be a key regulator of the activity of the protein. Further work will have to focus on the exact mechanisms of how p62 specifically interacts with HCMV proteins.

## Figures and Tables

**Table 1:** Posterior error probability (PEP) and modification localization probability.

Protein	Site	PEP Value	Localization Probability
p62	S262	6.58 × 10^−180^	0.999972

## Data Availability

Data are contained within the article.
